# Impact of an Electronic Health Service on Child Participation in Pediatric Oncology Care: Quasiexperimental Study

**DOI:** 10.2196/17673

**Published:** 2020-07-28

**Authors:** Britt-Mari Gilljam, Jens M Nygren, Petra Svedberg, Susann Arvidsson

**Affiliations:** 1 School of Health and Welfare Halmstad University Halmstad Sweden

**Keywords:** cancer, child care, communication, eHealth, patient participation

## Abstract

**Background:**

For children 6-12 years old, there is a shortage of electronic Health (eHealth) services that promote their participation in health care. Therefore, a digital communication tool, called Sisom, was developed to give children a voice in their health care. Children with long-term diseases want to be more involved in their health care and have the right to receive information, be listened to, express their opinions, and participate in decision making in health care. However, the outcomes of using Sisom in practice at pediatric oncology clinics have not been investigated.

**Objective:**

The aim of this study was to investigate children’s participation during appointments with pediatricians at pediatric oncology clinics, with or without the use of the eHealth service Sisom.

**Methods:**

A quasiexperimental design with mixed methods was used. We analyzed 27 filmed appointments with pediatricians for 14 children (8 girls and 6 boys) aged 6-12 years (mean 8.3 years) with a cancer diagnosis. The intervention group consisted of children who used Sisom prior to their appointments with pediatricians at a pediatric oncology clinic, and the control group consisted of children who had appointments with pediatricians at 4 pediatric oncology clinics. Data from observations from the videos were quantitatively and qualitatively analyzed. The quantitative analysis included manual calculations of how many times the pediatricians spoke directly to the children, the proportion of the appointment time that the children were talking, and levels of participation by the children. For the qualitative analysis, we used directed content analysis to analyze the children’s levels of participation guided by a framework based on Shier’s model of participation.

**Results:**

Pediatricians directed a greater proportion of their discussion toward the child in the intervention group (731 occasions) than in the control group (624 occasions), but the proportion of the appointment time the children talked was almost the same for both the intervention and control groups (mean 17.0 minutes vs 17.6 minutes). The levels of participation corresponded to the first three levels of Shier’s participation model: children were listened to, children were supported to express their views, and children’s views were taken into account. The results showed an increased level of participation by the children in the intervention group. Several codes that were found did not fit into any of the existing categories, and a new category was thus formed: children received information.

**Conclusions:**

This study shows that the eHealth service Sisom can increase children’s participation during appointments with health care professionals. Further studies employing a randomized control design focusing on the effects of eHealth services on children’s health outcomes, perceived participation, and cost-effectiveness could make a significant contribution to guiding the implementation of eHealth services in pediatric care.

## Introduction

### Background

Advancements in electronic Health (eHealth) services to promote health, participation, and communication have been made in recent years [1-4]. These are in the form of mobile health technology (mobile devices) [5], and the majority are aimed at adults and adolescents [6,7], while the range of eHealth advancements for younger children is limited [8]. Most eHealth services for adolescents do not focus on participation, are not validated, and lack proof of effectiveness [6,9-11]. Existing eHealth services instead primarily focus on self-management [6,10-12], symptom assessment [9,12,13], social support, support for self-care [12,13], or medication adherence [11,13,14]. Only one study on an eHealth service promoting participation for young children, 3-5 years old, has been performed [15], while there are no studies that describe an eHealth service that promotes participation for children 6 years old and older [16,17].

### Children’s Participation in Health Care

Children with long-term diseases such as cancer want to be have greater involvement in their own health care [18-21].They want to receive more information about emerging symptoms, treatment, and prognosis [22] and have the possibility to decide whether and to what extent they want to participate in decision-making processes regarding their own health care [18,23]. It is, however, difficult for children to explain how their long-term disease makes them feel [24] and to accept and manage the consequences and lifestyle related to their long-term disease [17]. It is thus essential to include children in their own health care and support them in their self-management responsibility in order to improve their wellbeing and reduce fear and anxiety [25] as well as to strengthen their self-confidence and independence [17,18,26]. Even if children’s participation is considered an essential part in health care, there is still a number of barriers to overcome in relation to health care professionals’ attitudes and viewpoints [27]. Furthermore, it is emphasized in the United Nations Convention on the Rights of the Child [28] that children have the right to be involved in all matters affecting them. Despite the knowledge that children want to be more involved in their health care, that their participation has positive effects on treatment and health outcomes, and that laws and regulations emphasize the requirement of patient participation in health care, there is still a lack of knowledge, strategies, and methods for strengthening children’s participation in pediatric health care and how to accomplish their participation [29,30].

### eHealth Services: Example of Sisom

**Figure 1:**
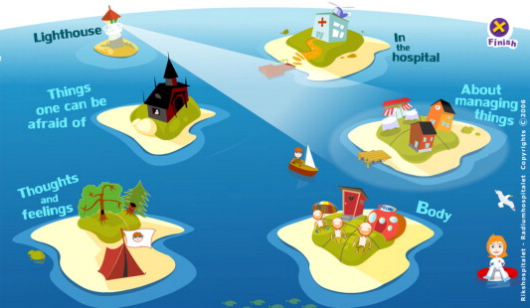
The 5 islands of Sisom.

Sisom is an eHealth service helping children to communicate in health care by engaging them in a playful virtual world [31,32]. Sisom was developed with a participatory design that included children with cancer in the process [31]. Previous research conducted on Sisom has primarilyfocused on usability and content validity, resulting in different diagnosis-specific versions and country-specific versions: Norway, Sweden, United States, and Canada [33-36]. In Sisom, the children travel by boat in an archipelago of islands with different subjects where they can express their feelings and symptoms by answering a set of 82 questions (Multimedia Appendix 1) [33]. The 5 islands represent different topics for the questions: (1) to handle things, (2) my body, (3) thoughts and feelings, (4) things you may be afraid of, and (5) in the hospital (Figure 1). The intent is that the children answer the questions in Sisom prior to a health care appointment or when they are hospitalized. A report of the children’s responses can be printed. Based on the children's answers, the health care professionals can talk to the children about how they perceive their situation and how they think about different health care–related and social issues. It has been shown that communication between children, their parents, and health care professionals is improved when using Sisom [37,38].

It has been highlighted that participatory approaches where patients have a real influence on the development of eHealth interventions lead to a more user-friendly and effective intervention [39]. This is especially important for interventions aimed at children, since adults lack a child’s perspective. However, children’s participation in the development of eHealth services is not common [40]. The use of eHealth services to enable and support children’s participation in pediatric health care is scarce. We found only one review of eHealth services that was designed to support communication between children with cancer and health care professionals with the purpose of strengthening children’s participation in care [12]. The implementation of eHealth services for children with chronic conditions is primarily focused on changing health behavior [7,14,41,42] and medical adherence [14], and most of the eHealth interventions that have been implemented in pediatric oncology clinics target emotional distress, health behaviors, health outcomes, and neurocognitive functioning [7], with insufficient evidence of effects. Furthermore, there is a need for more rigorous research and evaluations to determine eHealth service efficacy [6,7,14,42,43] especially in relation to eHealth services that are directed towards strengthening children’s participation in care. There is a lack of evidence to support that eHealth services actually promote children’s participation in their health care. It is also unclear to what extent children’s participation is supported and promoted in practice in pediatric oncology clinics. Thus, the aim of this study was to investigate children’s participation during appointments with pediatricians at pediatric oncology clinics, with or without the use of the eHealth service Sisom.

## Methods

### Design

**Figure 2:**
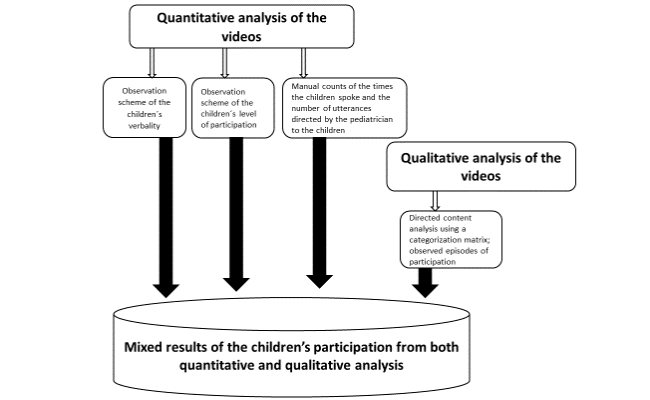
Processes for quantitative and qualitative analysis.

A quasiexperimental design [44] with a convergent parallel mixed method [45,46] was used, where the frequencies and differences between groups were calculated with descriptive statistics and qualitative content analysis was used to determine how children’s participation was manifested [47] (Figure 2).

### Participants

Inclusion criteria for participation were children 6-12 years old undergoing cancer treatment at 4 pediatric oncology clinics in Norway. Exclusion criteria were children who did not speak Norwegian, diagnosis of brain tumors, mental disability, developmental delay, speech disorders, or hearing impairments. The demographic characteristics are provided in Table 1.

**Table 1:** Demographic characteristics of the participants.

Characteristics		Intervention group, n (%)	Control group, n (%)
**Age (years)**			
	6-8	2 (33)	5 (63)
	9-12	4 (67)	3 (37)
**Gender**			
	Girl	2 (67)	6 (75)
	Boy	4 (33)	2 (25)
**Diagnosis**			
	Acute lymphoblastic leukemia (ALL)	3 (50)	3 (38)
	Lymphoma	1 (17)	2 (25)
	Carcinoma	1 (17)	0 (0)
	Not defined	1 (16)	3 (37)

### Recruitment

The children were successively recruited after receiving a cancer diagnosis and began the treatment. The head nurse identified eligible children and asked them and their parents if they were willing to be contacted about the study. The project assistant then informed the child and the parents, both verbally and in writing, about the content of the study including the voluntary nature of participation and the possibility to withdraw later in the process and asked if they were willing to participate. Families interested in participating signed an informed consent form. Of the 20 children who were asked to participate, 15 chose to opt-in. One child, who had disease-related complications, withdrew, resulting in 14 participants aged 6-12 years, of whom 6 were boys and 8 were girls.

### Data Collection

Data were collected through video recordings of the appointments with the 15 pediatricians, who conducted 1-5 recorded appointments each, for a total of 27 video-recorded appointments in outpatient examination rooms. Although an additional 3 pediatrician appointments were planned to be filmed with each child, this was not possible for all the children due to medical complications or relocations to another hospital. All the control group appointments took place prior to Sisom being introduced at the hospitals. The group of children in the intervention then met the pediatricians after Sisom was introduced. The appointments, both for the children in the control group and children in the intervention group, were in the form of routine meetings, which started with a nurse taking standard measurements of length, weight, and pulse oximetry. The pediatrician started by reviewing the children’s medication, reviewing infection status, and explaining the results of blood tests, supplemented by a medical examination. All those participating in the appointments were aware that they would be filmed. The children in the intervention group used Sisom while they were in the waiting room prior to meeting the pediatrician, and the project assistant was available for help. A report of the children’s responses was then printed and given to the pediatrician as well as to the parent and child; then, the meeting with the pediatrician took place. It was only during one appointment that the pediatrician revealed (to the researcher analyzing the recording) that Sisom had been used using the words: “I saw in your schedule that one of your feet is hurting; is it?” The word Sisom was not mentioned during any appointment.

### Data Analysis

The analysis was performed using a convergent parallel mixed method [46] to combine qualitative and quantitative data to be able to attain a more complete understanding [48] of the children’s participation during the appointments with pediatricians, with or without using Sisom. The quantitative and qualitative data were collected concurrently, and the results were combined and compared for convergence and differences [46]. All the video recordings were viewed several times with the aim of trying to gain an overall picture and see and hear everything that occurred during the appointments. The analysis process was carried out by two researchers who had no knowledge about which recordings were from the appointments during which Sisom had been used. The data acquisition consisted of manual counting and observations in accordance with an observation scheme and a categorization matrix (Table 2).

**Table 2:** Summary of the research questions, data collection methods, and data analysis.

Research questions	How many times did the pediatricians speak directly to the children?	How long did the children speak?	Which levels of participation were achieved during the appointments?	Which levels of verbality were achieved in the appointments?	How did the children’s participation manifest itself?
Data collection	Manual counting of the number of times the pediatricians spoke to the children.	Manual counting of the amount of times the children spoke.	Observations in accordance with two observation schemes with 4-degree scales were developed, and the children’s participation was ranked.	Observations in accordance with two observation schemes with 4-degree scales were developed, and the children’s level of verbality was ranked.	Observations according to a categorization matrix were developed and used for the collection of brief episodes of participation in the films.
Data analysis	Quantitative analysis	Quantitative analysis	Ranking	Ranking	Qualitative analysis

### Quantitative Analysis

Frequencies and differences were calculated using quantitative analysis. The number of times the pediatrician spoke directly to the child was counted manually and recounted repeatedly. Frequency calculations were performed to calculate the percent values of the proportion of the total appointment time that the child was talking, the proportion of the time the child spoke to the pediatrician, and the proportion of the total number of times the pediatrician spoke that was addressed to the child [49]. The number of times the children spoke and the number of times the pediatrician spoke to the children were calculated. Descriptive statistics of the results were compiled and divided into the intervention group and control group, after the blinding was revealed.

An observation scheme was used to assess the children's verbality [50]. The level of the children’s verbality during the appointments was ranked with a 4-degree verbality scale: (1) verbally inactive, gave <3 answers during the appointment and did not comment on anything or ask any questions; (2) limited verbality, gave >3 answers but no comments or questions; (3) moderate verbality, answered questions with ≥4 words but made <3 comments or questions; 4) verbally active, responded extensively to >3 questions, commenting or asking questions >3 times [50].

An observation scheme was developed by the authors to assess the children’s level of participation. The observation scheme was based on the levels in Shier's participation model “Pathways to participation,” a theoretical model intended to be used in practice when working with children up to 12 years old. The model consists of 5 levels: (1) children are listened to, (2) children are supported in expressing their views, (3) children’s views are taken into account, (4) children are involved in decision-making processes, and (5) children share power and responsibility over decision making [51]. The 3 lowest levels present levels of participation that are the limit of what is stated in the children’s Convention on the Rights of the Child as the meaning of participation. The 2 highest levels thus represent higher participation and are also consistent with the definition of shared decision making. The aim of the observation scheme was to rank the levels of the children’s participation during the pediatrician appointments, in relation to Shier’s 5 levels of participation. We defined in the observation scheme that the decision making should consider decisions concerning medication, caring, or procedure. To make the grading clear and understandable at each level, a 4-degree scale was used for each level in the observation scheme: (0) on a low level or not at all, (1) on a fairly low level, (2) on a high level, and (3) on a very high level (Multimedia Appendix 2). For example, at the first level of participation (children were listened to), “on a low level (0)” was assessed if the health care professionals only spoke to and listened to the parents during the conversation and if the health care professionals only took the initiative to ask the child on a few occasions or not at all. “On a fairly low level (1)” was assessed if the health care professionals spoke to and mostly listened to the parents and took the initiative to ask the child on some occasions but did not listen to what the child had to say. “On a high level (2)” was assessed if health care professionals spoke to and mostly listened to the child and took the initiative to ask the child something on several occasions but did not listen clearly to what the child was saying. “On a very high level (3)” was assessed if the health care professionals spoke to and mostly listened to the child and took the initiative to ask the child something on several occasions and listened clearly to what the child was saying. The grading for all levels is described in detail in Multimedia Appendix 2.

Face validity and content validity were assessed through the training of coders [52] to test the observation scheme and its reliability; two of the researchers analyzed the same video separately. Then all the researchers evaluated and made adjustments to the observation scheme. Interrater reliability was assessed in the next step [44], where a number of video recordings were examined by two of the researchers, who then compared their individual documentation.

### Qualitative Analysis

In order to attain a greater understanding of the results, a directed, deductive qualitative content analysis was performed to discern how the children’s participation manifested itself during the appointments [47]. A categorization matrix was developed from Shier’s participation model [51], with which the data from the videos were reviewed for content, passages of participation, and brief episodes of participation [47]. The analysis process was performed in the following steps: (1) looking at the videos and highlighting all episodes that, on first impression, appeared to represent passages of participation; (2) coding all highlighted passages using the predetermined categories in Shier’s model; and (3) giving a new category label that captures the essence of participation to any codes that could not be categorized with the predetermined categories [47]. Brief episodes in the films were found during the analysis of the video recordings where the children were actively excluded from participation. These film episodes were also included in the results as negative codes in order to more clearly describe the degree of the children’s participation. The blinding was released when the categorization process was completed, and the codes in each category were divided into the intervention group and control group. Two researchers completed the analysis, and to enhance the credibility and the dependability of the analysis, the data analysis was discussed continuously with all authors.

### Ethical Considerations

The videos were collected between 2006 and 2009 by the Center for Patient Involvement and Collaboration Research, a research group at Oslo University Hospital. This study was approved by the Regional Ethical Review Board in Oslo (2014/1599), with approval for use of the collected videos. The research group at Oslo University Hospital offered the data to the first author to use for this study. The difficulties in doing research on already collected data and to have to depend on the specific setting and design of the data collection were discussed by the authors as a limitation of the study. However, not analyzing already collected data could be considered unethical, which was considered in the decision to conduct this study. The participating children had approved their participation and expected that their contribution to the research would improve care for children with cancer. The funders Region Halland and the Center for Research on Welfare, Health, and Sport at Halmstad University had no access to the study data and have not influenced the way in which the data were analyzed.

## Results

### Sample

In the intervention group, 13 appointments with pediatricians were performed at 1 pediatric oncology clinic, with 2 girls and 4 boys participating (mean age 9.5 years). In the control group, 14 appointments with pediatricians were performed at 4 pediatric oncology clinics, with 6 girls and 2 boys (mean age 8.1 years) participating who received care as usual.

### Quantitative Results

#### Verbality

The appointments lasted from 6 to 41 minutes. The average times that the children and the parents met the pediatricians were 17.8 minutes for the intervention group (out of 231 minutes and 13 appointments) and 17.6 minutes for the control group (out of 246 minutes and 14 appointments; Multimedia Appendix 3). The children spoke to the pediatricians, on average, 1.14 minutes in the intervention group and 1.78 minutes in the control group (Multimedia Appendix 3). Except for answers to specific questions, the children responded by saying “yes,” “no,” or “hmmm” in most of the appointments. Short and unclear expressions were difficult to record and thus not included. The pediatricians in the intervention group spoke directly to the children on more occasions and for longer total time (average 56.23 minutes from 731 occasions) compared with the control group (average 44.57 minutes from 624 occasions; Multimedia Appendix 4). The children´s verbality was slightly higher in the control group than in the intervention group (Multimedia Appendix 4). 

#### Participation

There was a higher score for the children’s participation, based on Shier’s participation levels [51], when using Sisom compared with the control group. The higher scores are particularly noticeable for the following 3 levels: children were listened to, children were supported in expressing their views, and children’s views were taken into account (Table 3).

**Table 3:** The ranges (0-3) of the levels of children’s participation during the appointments with the pediatricians, according to Shier’s model.

Group and code number of the child		5 levels in Shier’s model of participation^a^				
		Children were listened to	Children were supported into expressing their views	Children’s views were taken into account	Children were involved in decision-making processes	Children shared power and responsibility over decision making
**Intervention group**						
	I.1	3	3	0	0	0
	I.1	3	3	0	0	0
	I.1	3	3	0	0	0
	I.2	3	3	0	0	0
	I.3	3	3	0	0	0
	I.3	2	3	0	0	0
	I.3	3	3	3	0	0
	I.4	3	3	3	3	0
	I.4	3	3	3	0	0
	I.4	3	3	3	3	0
	I.5	3	3	3	0	0
	I.6	3	3	3	0	0
	I.6	3	3	3	3	0
**Control group**						
	C.1	3	3	0	0	0
	C.1	3	1	0	0	0
	C.2	0	0	0	0	0
	C.3	0	0	0	0	0
	C.3	0	0	0	0	0
	C.4	0	0	0	0	0
	C.5	1	0	0	0	0
	C.6	1	1	1	0	0
	C.7	3	3	3	0	0
	C.7	1	0	0	0	0
	C.7	3	3	3	0	0
	C.8	1	0	0	0	0
	C.8	3	3	3	0	0
	C.8	3	3	3	0	0

^a^To grade the level of children’s participation, we used a 4-degree scale: (0) on a low level or not at all, (1) on a fairly low level, (2) on a high level, and (3) on a very high level.

### Qualitative Results

The findings from the directed qualitative content analysis showed passages and episodes of participation that were in line with the first 3 levels in the model presented by Shier: (1) children were listened to, (2) children were supported in expressing their views, and (3) children’s views were taken into account. However, the analysis of the videos did not reveal any passages or episodes that could be included in the fourth or fifth level of participation. One new category, which was not included in Shier’s model, named “children received information” emerged in the analysis (Table 4).

**Table 4:** Analysis of the videos: examples and number of codes in each group.

Group		New category	5 levels in Shier’s model of participation				
		Children received information	Children were listened to	Children were supported in expressing their views	Children’s views were taken into account	Children were involved in decision- making processes	Children shared power and responsibility for decision making
**Intervention group**							
	Examples	The pediatrician informed about what would happen. The pediatrician informed the child about a medicine.	The pediatrician gave the child time and waited for an answer. The pediatrician showed through body language and position that the child was in focus. The parent answered on behalf of the child.	The pediatrician asked the child a medical question in order to get the child’s perspective. The pediatrician asked if the child wanted to talk about something special.	The pediatrician followed the child’s wishes about the medical examination.	The child was involved in a decision about a blood test.	None
	Number of positive codes	84	21	100	2	1	0
	Number of negative codes	0	4	4	0	0	0
**Control group**							
	Examples	The pediatrician informed the child about the medical examination. Information was only directed to a parent.	The pediatrician talked over the head of the child. Children asked and said something without receiving attention from health care professionals.	The child expressed a desire on a medical aspect. The pediatrician asked the child about symptoms.	The physician followed the child’s wishes about the medical examination.	None	None
	Number of positive codes	36	5	32	3	0	0
	Number of negative codes	6	34	28	0	0	0

#### Children Received Information

Receiving information meant that the children were provided with medical information about what the pediatricians were doing and what could be expected to happen. The children also received information in response to their direct questions about, for example, test results, whether they could eat before a specific examination, or whether a peripheral venous catheter was required before an operation. In the intervention group, the pediatricians gave the children information about examinations and medication during the appointments. The disease-related information was often repeated several times during the same appointment, and almost all the children were informed by the pediatrician about the medical examination, what the pediatrician would do, and the result of the examination. The children were also often informed about the disease, symptoms, and side effects. Some children received health advice, such as the importance of eating so that the body gets nutrients. No child from the intervention group asked for further information during the appointments, but in the control group, the children asked for information on a number of occasions, such as “Why did you press there?” (when the pediatrician palpated his stomach; boy, 6 years old, control group).

The information the children in the control group asked for during the appointments was whether the child had to spend Christmas at the hospital or about examinations, tests, their anatomy, and things they did not understand. One exception to this was one child in the control group who was informed about the course of the disease and the future. The explanation during this appointment was given slowly and in a language adapted to the child's level of development. During another appointment in the control group, the pediatrician had two accompanying medical colleagues. The conversation during this appointment was conducted in English and was thus not understandable to the Norwegian-speaking child and was only directed to the medical colleagues. The pediatrician informed his colleagues about what he did in English during the medical examination but said nothing to the child. The pediatricians informed the children about the results of the medical examination and the medicines and their side effects in only a few of the appointments in the control group, and the information was aimed only at the parents.

#### Children Were Listened To

The children were listened to when the pediatrician paid full attention to the child and gave the child time to think before answering. The pediatricians often turned to the children during the conversation in the appointments in the intervention group. The pediatricians’ body language, sitting close to the child and looking at the child, showed that they were really listening. The pediatricians sat either squatting or near the child and thus had better eye contact.

The pediatricians waited for and gave the child time to think before answering when talking with the children during the appointments in the intervention group. The pediatricians listened to the children’s answers and asked further questions or confirmed that they were listening with an “Hmm.” The child’s response was followed up by the pediatrician in the cases when parents and children responded differently to the same question. One physician moved closer to the child to get better eye contact and repeated the questions after the parent repeatedly responded on behalf of the child. For example, a pediatrician asked “Doesn’t the food taste good anymore?” When there was no answer from the child, the pediatrician squatted twice in order to get better eye contact with the boy, pats him on the blanket, and repeats the question (boy, 7 years old, intervention group).

The children were not listened to when the pediatrician spoke quickly without giving the child time to reflect and answer or when the pediatrician used language that was difficult for a child to understand. These types of communication occurred during many appointments in the control group, although there were also appointments when the pediatrician listened to the child. The pediatricians were more often focused on the parents than on the child during the appointments in the control group. The pediatricians looked quickly at the parent and seemed to expect the parent to respond even though the question was initially addressed to the child. The conversation between the child and pediatrician was performed through a parent, leaving the child ignored during some of the appointments in the control group. On one occasion, the child was playing with the blood pressure equipment and said he would like to take his blood pressure, but none of those present, including the pediatrician, nurse, and parent, responded or reacted. On one occasion, when another pediatrician asked a child what medications the child took, the latter began to respond, but the pediatrician did not listen to the child and turned to the mother instead. The child got upset and went and put on music at a high volume. During other appointments, the pediatrician asked the parents how the child was feeling, even though the child was sitting next to them. One pediatrician even expressed his desire for the child to be quiet. Several children during the appointments in the control group said or asked things without anyone listening and responding. On one occasion, a child started talking to her mother, and the pediatrician drowned the child by telling her mother a story about his private holiday trip. This child protested loudly without any reaction from the pediatrician or support from the mother.

#### Children Were Supported in Expressing Their Views

The children were supported in expressing their views, which entailed the child being asked and invited into the conversation, but also that the children themselves initiated discussions about for example, how the child wanted the procedure or process to be conducted during a medical examination or that the child wanted to return home. The children often appeared to be tired and silent in both the intervention and control groups; thus, support from the pediatrician was needed. The children were often invited into the conversation during the intervention group’s appointments. The pediatricians were attentive in their interactions with the child and gave the latter time to express themselves, which in some appointments gave the children courage and capability to express their opinions. The pediatrician offered their support and built trust by trying to get the children to talk about their problems, thoughts, and reflections. Some pediatricians asked the children questions about their social life, friends, social exclusion, and mental health, while others only asked questions about the child’s disease and treatment. The pediatricians had nearly all their attention directed to the child during some of the appointments in the intervention group. Although some children were mostly silent, they were then invited into the conversation through questions directed at them and being given enough time for answering and expressing their views. The conversations and questions during the various appointments mainly concerned the children's condition and possible symptoms. There were often open questions posed by the pediatricians.

When the parents in the intervention group said, during one appointment, that their child had a stomachache, the pediatrician turned to the child and asked questions about it. During some of the other appointments in the intervention group, the pediatricians asked how the children wanted the medical examination to be carried out, and sometimes the children expressed their views spontaneously. A girl said that she preferred to lie down during the examination. The children in the intervention group were often directly asked questions about medication, such as which medicines they took. The problems the children spoke about were, for example, pain when measuring blood pressure and a medicine that was only available as a tablet that the child had difficulties in swallowing. The children often demonstrated their knowledge about medicines; for example, they could list all the medicines they took and often the doses. Moreover, they spoke about incidents such as infections and blood transfusions since the last appointment, as well as new symptoms, such as bruises. For example, a 9-year-old girl in the intervention group said “The medicine is a bit awkward; we have to crush it and mix it with yoghurt.

The pediatricians offered emotional support to the children during many of the appointments in the intervention group by encouraging them to come to them if problems arose. The pediatricians generally wanted to know if the children had difficult or unpleasant experiences or if they had other questions and concerns. Several of the pediatricians explained to the children that they wanted to understand how the children thought and felt. A pediatrician explained at one appointment that it was sad but normal that the child could no longer ride a bike or ski downhill, but that it would be possible to do it again in the future, while another pediatrician praised the child’s strength in showing her baldness despite everyone in school knowing and being able to see. The pediatricians also asked open questions about participation where they offered support to the children by allowing them to express their views: “Is there anything you have forgotten to ask about or anything else you want to say?” (pediatrician to girl, 9 years old, intervention group).

The pediatricians rarely invited the children into the conversation during the control group’s appointments; for example, at one appointment, many questions were asked very quickly, not giving the child time to answer. The pediatricians gave instructions rapidly during the medical examination in some of the control group’s appointments, not allowing the children to express their opinions.

Talking about private things helped the children relax and feel secure. The pediatrician talked to the children about private things in approximately half of the appointments in the intervention group. They talked about how the children spent the summer, whether they had swum, and whether they would be going away on holiday. Other subjects were also raised, such as being unable to go to school, difficulties in sports activities, and talking about computer games and Christmas presents. The pediatrician only talked to the children about private things, such as pets and school, in a few of the control group’s appointments.

#### Children’s Views Were Taken Into Account

The level in Stier’s model concerning the children’s views being taken into account pertains to the pediatricians’ consideration of the children’s views. Only a few brief episodes in the videos were found for this level. The children’s views were taken into account during the interventions group’s appointments. For example, at one appointment when a child asked her parent if she could go out and listen to music, it was noted by the pediatrician who said to the child that she could go out if that was what she preferred, and at another appointment, a child expressed a wish to lie down during the medical examination.

You can do it [the examination] when I’m lying down, that‘s better.

girl, 9 years old, intervention group

Jump up and lie down then [on the examination table].

pediatrician

A child in the control group was informed during an appointment of the need to stay and have treatment, and the child became very upset. The child's opinion was taken into account in a respectful and supportive way when the child started to scream and cry. The pediatrician, nurse, and parents were completely silent, allowing the child to express her disappointment. The adults showed empathy through eye contact and body language expressing that they were also sad on behalf of the child. A child protested about having to remove clothes during another appointment in the control group. This child's opinion was taken into account by the pediatrician who lifted the clothes up a little instead.

#### Children Were Involved in Decision Making

The children were not involved in decision making in their health care. The pediatricians made most of the decisions, purely medical, both in the control and intervention groups, and neither the parents nor the children participated in these decisions. The decisions concerned continuation or temporary suspension of treatment, a need for blood transfusion, removal of a subcutaneous venous port, and planning for new blood tests and were expressed as already having been determined. Only one brief episode was found, which was in the intervention group, where a nurse suggested that the child allowed blood samples to be taken prior to talking to the pediatrician, and the latter then asked the child if this was a good idea. The child participated in the decision by deciding whether blood samples could be taken prior to or after the appointment with the pediatrician.

#### Children Shared Power and Responsibility for Decision Making

No brief episode was found showing that children shared power and responsibility for decision making.

## Discussion

### Principal Findings

The overall results showed increased participation for the children in the intervention group using Sisom compared to the control group, especially in relation to the lower levels of participation (levels 1-3) in Shier’s model [51]. Levels 4 and 5 concern the child’s possibilities for sharing decision making and responsibility with health care professionals. These levels were barely noticeable in any of the appointments in the two groups. These findings confirm previous research that has shown that children’s possibilities for participating in decisions that concern them in health care are almost nonexistent [50,53,54]. It has been highlighted both in research and clinical practice that there is a need for guidelines, strategies, and methods to enable a higher level of children’s participation in clinical practice. This study showed that the use of the eHealth service Sisom can increase the health care professional’s ability to promote children’s participation, but there is much more to do to reach a higher level where the children are co-actors and share decisions about issues in their care. To change and restructure the working procedures for children’s participation in practice, it is crucial to consider both the individual and contextual factors such as attitudes, values, knowledge, readiness for behavioral change, culture, resources, and priorities [55]. For example, it has been maintained that pediatric care needs guidance and support [56], education [57], training [58], and interventions and methods (for example, eHealth services such as Sisom) [29,53,59,60] to increase children’s participation. This entails extensive effort by both the health care services provided for children and the professionals who work there to increase their ability to involve children in decision making. It is also necessary for them to access adapted and person-centered information for children to have the ability to participate in decisions [19,20]. The children in the intervention group received more information from the pediatrician, expressed in a child-centered way and adapted to the needs of the child, in comparison with the control group. However, in the control group, the children asked for more and further information than those in the intervention group. One explanation for these differences between the groups could be that the pediatricians in the intervention group were more focused on and paid greater attention to the child’s problems expressed through the use of Sisom. The pediatricians thus provided information based on the children’s perspectives. However, information in pediatric health care is a complex aspect. The children’s preference for and ability to absorb information could vary depending on different factors such as personality, age, maturity, and disease state [20]. Another factor could be that the child is afraid of appearing to be stupid or feeling too shy to ask for information [61]. The child’s preferences could also vary in different situations and change over time [23,28,62,63]. Previous research has shown that children speak infrequently to health care professionals during pediatric appointments [64]. Information in pediatric health care has also been highlighted by children with different diseases as being important for their possibilities for participating in health care [65,66]. The directed qualitative analysis in our study revealed one further category termed “children received information,” which is not included in Shier’s model [51]. The health care professionals were giving the children information with the purpose of letting them know and understand what would be happening and to experience control over the situation.

It appears that health care professionals find it more difficult to give children space and opportunity to speak during appointments without having the support from an eHealth service such as Sisom. The children can convey problems and thoughts through Sisom, which can provide the health care professionals with a different way to engage with the children and to facilitate interaction. The compelling ambition, as emphasized in the United Nations Convention on the Rights of the Child [27], is that pediatric care and professionals’ interaction with the child should lead to the children being listened to and supported in expressing their views, these views being taking into account, and children being involved in the decision-making processes [51]. The decision-making level was not, however, achieved in this study. Even in care situations where there is a strict treatment protocol that needs to be followed in order to succeed with the care, informing and interacting about the treatment and care plan with the child are important for the child to understand, be able to ask questions [19], and express how they want to be involved in decision making [19,23]. The pediatricians in the intervention group were more focused on the children and seemed to be more aware of their own behavior when interacting with the child, which is important for the children to have positive experiences with care [67,68]. The use of an eHealth service for children has the potential of facilitating transition towards more trustful and supportive behavior from health care professionals [37,38,69,70].

### Methodological Considerations

The results in this study would have shown higher levels of participation for the children in the intervention group if based solely on the quantitative methodology, but the reasons for these results and how they came about would have remained unknown. Supplementing the methodology with a qualitative approach helped to gain an understanding of how the eHealth service, Sisom, can influence health care professionals through the visualization of the children’s needs and promotion of interactions, thus demonstrating the benefit of a mixed method approach [48].

Video observations are used in studies to observe how people behave towards each other, how they act and interact, to see body language and glances, and to hear what they talk about [71]. A video-recorded observation can be preferred in complex processes, such as appointments with pediatricians in health care settings, where many different things can happen simultaneously, and the researcher is able to review the videos several times [72]. It was sometimes difficult to see facial expressions and hear who was talking and what they were saying in the analyzed videos, partly due to technical reasons but also due to the participants’ location in the room. This led to the videos being reviewed many times to ensure that the content was perceived correctly. The pediatricians’ behavior towards the children can vary based not only on the use of Sisom but also on the different personalities and attitudes of the pediatricians and the different clinics’ organization and working procedures. However, all pediatricians have specialist expertise and lengthy experience working and interacting with children. The children were between 6 and 12 years old, which means different levels of development and verbality that may have affected their interactions in the appointments. However, this was not obvious in relation to the children’s statements to the pediatrician or their verbalities. This may impair the study’s credibility as we draw conclusions based on the differences between the control and intervention groups. A strategy was applied to strengthen the credibility and dependability of the study, whereby the coders were trained and the similarity in the analysis of the two researchers was assessed by interrater reliability. Detailed information about the analysis process has been provided, and examples of citations in the qualitative results are presented for confirmation.

### Study Limitations

One limitation could be that the data were collected several years ago. The fact that the data were collected by other researchers led to difficulties in obtaining facts about the execution of this data collection. For example, it was unclear if the child had met the current pediatrician before. The sample size was too small to perform significant statistical calculations. It is possible that health care professionals are more child-centered today since the importance of this approach has received greater attention in recent years. However, even if we are aware of some limitations in this study, it is important to investigate the implementation of eHealth services such as Sisom in daily practice since there is still a lack of similar resources to promote children’s participation in health care. This study can thus make an important contribution.

### Conclusions

eHealth services, such as Sisom, can increase children’s participation in health care and influence health care professionals’ ways of communicating with children in health care services. The overall results showed increased participation for the children in the intervention group compared to the control group, but a higher level of participation where the child could share decision making and responsibility with health care professionals was not visible in any of the groups. Sisom provides opportunities for enhanced improvement in health care, but its capabilities are far from being fully utilized. Further studies employing a randomized control design focusing on the effects of eHealth services on children’s health outcomes, perceived participation, and cost-effectiveness could make a significant contribution to guiding implementation of eHealth services in pediatric care.

## Appendix

Multimedia Appendix 1 Examples of questions in Sisom.

Multimedia Appendix 2 Observation scheme of the children's levels of participation.

Multimedia Appendix 3 Statements during appointments in the intervention and control groups.

Multimedia Appendix 4 Verbality in the intervention and control groups.

## Abbreviations

eHealth: electronic health.
