# Supplementary material for: Impact of an Electronic Health Service on Child Participation in Pediatric Oncology Care: Quasiexperimental Study
Source: J Med Internet Res. 2020 Jul 28;22(7):e17673. doi: 10.2196/17673 (PMC7420525; doi:10.2196/17673)
Supplement: Multimedia Appendix 1 [file jmir_v22i7e17673_app1.docx]

**The 82 questions in Sisom**

**At the hospital**

- Difficult to take medicines
- Treatment is uncomfortable
- Taking out stitches hurts
- Getting an IV hurts
- Blood draws hurt
- Checking blood pressure hurts
- Central line dressing changes hurt
- Anesthesia feels awful
- Taking off a band-aid hurts
- Getting a tube feels awful
- Shots hurt

**My body**

- Show where you have pain or discomfort by choosing one of the tattoos. Choose the purple tattoo if you have bruises, the green tattoo if you have pain or the red tattoo if you have a rash. Use the tattoo to show where on the body the problem is.
- Can’t hold it when I have to pee
- Peeing hurts
- Pooping hurts
- Diarrhea
- Have to go to the bathroom all the time
- Lots of hair on my body
- Got no hair
- Fat
- Thin
- Feel sick
- Get cold easily
- Hot or sweaty
- Throwing up
- Often dizzy
- Pins and needles in my arms and legs
- Feeling clumsy
- Tired a lot
- Trouble breathing
- Stuffy nose
- Cough a lot
- Shaky hands
- Trouble walking or running
- Trouble hearing
- Eye problems
- Feeling my heart beating fast

**About managing things**

- Often thirsty
- Eating is difficult
- Drinking is difficult
- Things taste or smell different
- Want to eat often
- Can't follow when others talk
- Can’t do anything for very long
- Forget things
- Reading and writing is difficult
- Don’t learn as much as the others
- Concentrating is hard
- Sleeping problems
- Relaxing is difficult
- To do things by myself is difficult
- Need help washing and getting dressed
- Get tired quickly

**Thoughts and Feelings**

- Don’t know what I want
- Difficult to tell others how I feel
- Would like to be by myself more
- Miss home
- Would like to make my own choices more often
- Difficult to get answers to what I’m wondering about
- Believe others are upset because I’m ill
- Think that it’s my fault that I’m sick
- Cannot be with my friends as much as before
- Feel different than other children
- Miss my family and friends
- Tired of talking and nagging
- Feel lonely
- Act younger than I am
- Get angry easily
- Get sad easily
- Get embarrassed easily
- Feel stupid
- Nothing is fun anymore

**Things one might be afraid of**

- Afraid that the cancer might come back
- Afraid about getting sick from someone else
- Afraid that my hair won’t grow back
- Afraid that the treatment will make me more sick
- Afraid of being alone
- Afraid of maybe dying from my illness
- Afraid but don’t know why
- Nightmares
