# Supplementary material for: Impact of an Electronic Health Service on Child Participation in Pediatric Oncology Care: Quasiexperimental Study
Source: J Med Internet Res. 2020 Jul 28;22(7):e17673. doi: 10.2196/17673 (PMC7420525; doi:10.2196/17673)
Supplement: Multimedia Appendix 2 [file jmir_v22i7e17673_app2.docx]

| **Level of participation** | **Ranking**  0 = To a low extent or not at all  1 = To a fairly low extent  2 = To a high extent  3 = To a great extent |
| --- | --- |
| Children were listened to | 0 (To a low extent or not at all): Healthcare professionals were only directed and listened to the parents during the conversation. Took the initiative to ask the child on a few occasions or not at all.  1 (To a fairly low extent): Healthcare professionals were directed and mostly listen to the parents. Took the initiative to ask the child on some occasions, but did not listen to what the child had to say.  2 (To a high extent): Healthcare professionals were directed and mostly listened to the child. Took the initiative to ask the child something on several occasions, but did not listen clearly to what the child was saying  3 (To a great extent): Healthcare professionals were directed and mostly listened to the child. Took the initiative to ask the child something on several occasions, and listened clearly to what the child was saying. |
| Children were supported in expressing their views | 0 (To a low extent or not at all): Healthcare professionals were directed and only listened to the parents during the conversation. Took the initiative to support the child in expressing his or her opinions and views on a single occasion or not at all.  1 (To a fairly low extent): Healthcare professionals were directed and mostly listened to the parents. Took initiative to give the child support in expressing their opinions and views on some occasions, but did not listen to what they had to say.  2 (To a high extent): Healthcare professionals were directed and mostly listened to the child. Took initiative to give the child support in expressing their opinions and views on numerous occasions, but did not listen clearly to what the child was saying.  3 (To a great extent): Healthcare professionals were directed and mostly listened to the child. Took the initiative to give the child support in expressing their opinions and views on numerous occasions, and listened clearly to what the child was saying. |
| Children´s views were taken into account | 0 (To a low extent or not at all): Healthcare professionals were directed and only listened to the parents during the conversation. Took the initiative to respond to the child's opinions and views on any occasion or not at all.  1 (To a fairly low extent): Healthcare professionals were directed and mostly listened to the parents. Took initiative to respond to the child's opinions and views on some occasions, but did not listen to what they had to say.  2 (To a high extent): Healthcare professionals were directed and mostly listened to the child. Took initiative to respond to the child's opinions and views on numerous occasions, but did not listen clearly to what the child was saying.  3 (To a great extent): Healthcare professionals were directed and mostly listened to the child. Took the initiative to respond to the child's views and views on numerous occasions, and listened clearly to what the child was saying. |
| Children were involved in decision making | 0 (To a low extent or not at all): Healthcare professionals were directed and only listened to the parents during the conversation. Took the initiative to involve the child in decision-making processes on a few occasions or not at all.  1 (To a fairly low extent): Healthcare professionals were directed and mostly listened to the parents. Took initiative to involve the child in decision-making processes on some occasions, but did not listened to what they had to say.  2 (To a high extent): Healthcare professionals were directed and mostly listened to the child. Took initiative to involve the child in decision-making processes on numerous occasions, but did not listen clearly to what the child was saying.  3 (To a great extent): Healthcare professionals were directed and mostly listened to the child. Took initiative to involve the child in decision-making processes on numerous occasions, and listened clearly to what the child was saying. |
| Children shared power and responsibility over decision making | 0 (To a low extent or not at all): Healthcare professionals were directed and only listened to the parents during the conversation. Took the initiative to share power and responsibility over decision making with the child on a few occasions or not at all.  1 (To a fairly low extent): Healthcare professionals were directed and mostly listened to the parents. Took initiative to share power and responsibility over decision making with the child on a few occasions, but did not listen to what they had to say.  2 (To a high extent): Healthcare professionals were directed and mostly listened to the child. Took the initiative to share power and responsibility over decision making with the child on numerous occasions, but did not listen clearly to what the child was saying.  3 (To a great extent): Healthcare professionals were directed and mostly listened to the child. Took initiative to share power and responsibility over decision making with the child on numerous occasions, and listens clearly to what the child was saying. |
