# Supplementary material for: Impact of an Electronic Health Service on Child Participation in Pediatric Oncology Care: Quasiexperimental Study
Source: J Med Internet Res. 2020 Jul 28;22(7):e17673. doi: 10.2196/17673 (PMC7420525; doi:10.2196/17673)
Supplement: Multimedia Appendix 3 [file jmir_v22i7e17673_app3.docx]

| Appointments | Code number of the child | The age of the child, years | The time of the appointment, minutes | A total of the child´s statements, minutes (%) | Statements by the child to the pediatrician, minutes (%) |
| --- | --- | --- | --- | --- | --- |
|  |  |  |  |  |  |
| Appointments in the intervention group | I.1 | 11 | 9 | 0.60 (7) | 0.60 (7) |
|  | I.1 | 11 | 14 | 0.15 (1) | 0.01 (0) |
|  | I.1 | 11 | 20 | 0.50 (3) | 0.36 (2) |
|  | I.2 | 7 | 11 | 0.13 (1) | 0.13 (1) |
|  | I.3 | 8 | 11 | 0.43 (4) | 0.01 (0) |
|  | I.3 | 8 | 20 | 0.54 (3) | 0.12 (1) |
|  | I.3 | 8 | 12 | 0.33 (3) | 0.15 (1) |
|  | I.4 | 9 | 27 | 3.60 (13) | 2.38 (9) |
|  | I.4 | 9 | 24 | 2.10 (9) | 1.56 (7) |
|  | I.4 | 9 | 13 | 1.14 (9) | 0.88 (7) |
|  | I.5 | 10 | 33 | 0.30 (1) | 0.18 (1) |
|  | I.6 | 12 | 15 | 2.30 (15) | 2.23 (15) |
|  | I.6 | 12 | 22 | 2.70 (12) | 1.96 (9) |
|  |  |  |  |  |  |
| Mean |  | 9.5 | 17,8 | 1.14 (7) | 0.81 (5) |
|  |  |  |  |  |  |
| Appointments in the control group | C.1 | 7 | 14 | 0.68 (5) | 0.29 (2) |
|  | C.1 | 7 | 27 | 2.31 (9) | 0.75 (3) |
|  | C.2 | 11 | 39 | 3.10 (8) | 1.46 (4) |
|  | C.3 | 7 | 7 | 0.22 (3) | 0.13 (2) |
|  | C.3 | 7 | 11 | 1.20 (11) | 0.52 (5) |
|  | C.4 | 12 | 12 | 0.70 (6) | 0.56 (5) |
|  | C.5 | 6 | 22 | 2.58 (12) | 0.72 (3) |
|  | C.6 | 7 | 13 | 1.35 (10) | 0.65 (5) |
|  | C.7 | 9 | 14 | 1.00 (7) | 0.76 (5) |
|  | C.7 | 9 | 7 | 0.07 (0) | 0.05 (1) |
|  | C.7 | 9 | 23 | 3.00 (13) | 2.28 (10) |
|  | C.8 | 6 | 10 | 0.75 (8) | 0.38 (3) |
|  | C.8 | 6 | 6 | 0.45 (8) | 0.41 (7) |
|  | C.8 | 6 | 41 | 7.50 (18) | 2.23 (5) |
|  |  |  |  |  |  |
| Mean |  | 8.4 | 17.6 | 1.78 (10) | 0.80 (4) |
