# Supplementary material for: Impact of an Electronic Health Service on Child Participation in Pediatric Oncology Care: Quasiexperimental Study
Source: J Med Internet Res. 2020 Jul 28;22(7):e17673. doi: 10.2196/17673 (PMC7420525; doi:10.2196/17673)
Supplement: Multimedia Appendix 4 [file jmir_v22i7e17673_app4.docx]

| Appointments | Code number of the child | The age of the child (years) | A total of the pediatrician´s statements, number | Statements by the pediatrician to the child, number (%) | The child’s level of verbality  (range 1-4) |
| --- | --- | --- | --- | --- | --- |
|  |  |  |  |  |  |
| Appointments in the intervention group | I.1 | 11 | 61 | 53 (87) | 2 |
|  | I.1 | 11 | 61 | 39 (64) | 1 |
|  | I.1 | 11 | 96 | 51 (53) | 1 |
|  | I.2 | 7 | 82 | 56 (68) | 1 |
|  | I.3 | 8 | 42 | 24 (57) | 1 |
|  | I.3 | 8 | 82 | 41 (50) | 2 |
|  | I.3 | 8 | 55 | 39 (71) | 1 |
|  | I.4 | 9 | 107 | 97 (91) | 4 |
|  | I.4 | 9 | 103 | 76 (74) | 4 |
|  | I.4 | 9 | 59 | 39 (66) | 4 |
|  | I.5 | 10 | 145 | 102 (70) | 1 |
|  | I.6 | 12 | 60 | 53 (88) | 4 |
|  | I.6 | 12 | 78 | 61 (78) | 4 |
|  |  |  |  |  |  |
| Mean |  | 9.5 | 79.3 | 56.23 (71.2) |  |
|  |  |  |  |  |  |
| Appointments in the control group | C.1 | 7 | 55 | 27 (13) | 2 |
|  | C.1 | 7 | 113 | 80 (71) | 2 |
|  | C.2 | 11 | 133 | 79 (59) | 2 |
|  | C.3 | 7 | 38 | 15 (39) | 1 |
|  | C.3 | 7 | 47 | 10 (21) | 3 |
|  | C.4 | 12 | 67 | 48 (72) | 3 |
|  | C.5 | 6 | 87 | 49 (56) | 4 |
|  | C.6 | 7 | 50 | 29 (58) | 4 |
|  | C.7 | 9 | 90 | 58 (64) | 4 |
|  | C.7 | 9 | 31 | 16 (52) | 1 |
|  | C.7 | 9 | 87 | 62 (71) | 4 |
|  | C.8 | 6 | 42 | 10 (24) | 1 |
|  | C.8 | 6 | 47 | 38 (81) | 4 |
|  | C.8 | 6 | 190 | 103 (54) | 4 |
|  |  |  |  |  |  |
| Mean |  | 8.4 | 76.9 | 44.57 (57.9) |  |
|  |  |  |  |  |  |
